# Downregulation of TFPI in breast cancer cells induces tyrosine phosphorylation signaling and increases metastatic growth by stimulating cell motility

**DOI:** 10.1186/1471-2407-11-357

**Published:** 2011-08-17

**Authors:** Benedicte Stavik, Grethe Skretting, Hans-Christian Aasheim, Mari Tinholt, Lillian Zernichow, Marit Sletten, Per Morten Sandset, Nina Iversen

**Affiliations:** 1 Department of Medical Genetics, Oslo University Hospital, Oslo, Norway; 2 Institute of Clinical Medicine, University of Oslo, Oslo, Norway; 3 Department of Haematology, Oslo University Hospital, Oslo, Norway

**Keywords:** TFPI-1, adhesion, migration, invasion, tumor suppressor

## Abstract

**Background:**

Increased hemostatic activity is common in many cancer types and often causes additional complications and even death. Circumstantial evidence suggests that tissue factor pathway inhibitor-1 (TFPI) plays a role in cancer development. We recently reported that downregulation of TFPI inhibited apoptosis in a breast cancer cell line. In this study, we investigated the effects of TFPI on self-sustained growth and motility of these cells, and of another invasive breast cancer cell type (MDA-MB-231).

**Methods:**

Stable cell lines with TFPI (both α and β) and only TFPIβ downregulated were created using RNA interference technology. We investigated the ability of the transduced cells to grow, when seeded at low densities, and to form colonies, along with metastatic characteristics such as adhesion, migration and invasion.

**Results:**

Downregulation of TFPI was associated with increased self-sustained cell growth. An increase in cell attachment and spreading was observed to collagen type I, together with elevated levels of integrin α2. Downregulation of TFPI also stimulated migration and invasion of cells, and elevated MMP activity was involved in the increased invasion observed. Surprisingly, equivalent results were observed when TFPIβ was downregulated, revealing a novel function of this isoform in cancer metastasis.

**Conclusions:**

Our results suggest an anti-metastatic effect of TFPI and may provide a novel therapeutic approach in cancer.

## Background

An association between cancer and the hemostatic system has been recognized for almost two centuries [1-3]. Thrombosis is nevertheless still among the leading causes of death in cancer patients [3]. The coagulation cascade is triggered by tissue factor (TF) and results in the formation of a fibrin network. TF pathway inhibitor-1 (TFPI) is mainly known for its role in the hemostatic system where it is responsible for the inactivation of TF-induced coagulation [4,5]. There is, however, a growing body of evidence of a new role of TFPI in cancer. Elevated levels of plasma TFPI in cancer patients have previously been reported [6,7], and TFPI expression was demonstrated in several tumors, including breast cancer tissue and cells [8,9], indicating a possible involvement of TFPI in cancer biology [9].

Two main isoforms of TFPI are recognized, TFPIα and TFPIβ. TFPIα consists of three Kunitz-type domains and a basic C-terminus, and is either secreted or attached to the cell membrane through an unknown glycosylphosphatidylinositol (GPI) linked molecule. TFPIβ contains only the first two Kunitz-domains and has a different C-terminus with a GPI anchor that attaches it to the cell membrane [10-13]. The functional role of TFPIβ is not yet completely understood [14]. In a recent study, we demonstrated a pro-apoptotic effect of both TFPIα and TFPIβ in breast cancer cells *in vitro*, while corresponding downregulation of endogenous TFPI resulted in reduced apoptotic activity [15]. It has previously been reported that treatment of cancer cells with TFPIα, either recombinant or through ectopic overexpression, resulted in reduced primary and metastatic tumor growth and tumor cell adhesion in murine models [16,17]. The non-hemostatic activity of TFPIα has in several cases been reported to be dependent on the C-terminal part of the protein [18-23], and a peptide corresponding to the C-terminus of TFPIα has been shown to inhibit angiogenesis and tumor growth *in vivo *[24].

Requirements for malignant cancer cells to form metastases are to escape from the primary tumor, to passage through the circulatory system, and to establish secondary tumors at distant sites. Such movement requires abnormal growth and adhesion characteristics and penetration of the extracellular matrix (ECM) surrounding the tumor and vessel organs [25]. Integrins are cell surface receptors involved in adhering cells to the ECM and mediate cell movement [26]. Binding of the ligand mediates attachment to the matrix, while phosphorylation of the receptor relays spreading of the cells to the matrix [27] through the formation of filopodia, lamellipodia, and stress fibers [28]. Matrix metalloproteinases (MMPs) are proteases which are able to cleave the ECM and basement membrane and are thus important for metastasis of cancer cells [29]. In the present study, we investigated how downregulation of TFPI would affect the growth and metastatic abilities of human breast cancer cells *in vitro*, and the possible mechanisms involved. To investigate the significance of the C-terminal end of TFPIα, downregulation of only TFPIβ was conducted. We show here that lowered expression of both isoforms of TFPI or only TFPIβ induced cell signaling and self-sufficient growth, and stimulated adhesion, migration, and invasion of the cells. The increase in invasion was connected to elevated activity of the proteases MMP-2 and MMP-9.

## Methods

### Cell culture and stable cell lines

The intraductal breast carcinoma cells Sum102 were grown in Human mammary epithelial cell (HuMEC) ready medium (Invitrogen, Carlsbad, CA, USA) containing HuMEC supplements (epidermal growth factor, hydrocortisone, isoprotenerol, transferrin and insulin) and Bovine Pituitary Extract (Invitrogen). The human mammary adenocarcinoma cells MDA-MB-231 were grown in DMEM (Lonza, Viviere, Belgium) with 10% fetal bovine serum (FBS, Lonza). Cells were cultured at 37°C in an incubator with a humidified atmosphere and 5% CO_2_.

Stable cell lines with TFPI downregulated were established as previously described [15]. In short, double stranded cDNAs encoding shRNAs targeting both isoforms of TFPI were cloned into the retroviral vector pSiRPG [30] and co-transfected into HEK293T cells together with viral helper vectors. Sum102 and MDA-MB-231 wild type cells were infected with the viral particles and positive clones were selected by the use of puromycin. The stable cell line pools with TFPI downregulated were named pSiRPG-shRNA4, -6 and -7. For downregulation of TFPIβ, cDNAs encoding shRNAs against this isoform were cloned into the pSiRPG vector and transduced into MDA-MB-231 cells (shRNA sequences available upon request). The resulting stable cell line pools were named pSiRPG-shRNA7β and -9β. Cell lines transduced with an empty vector were used as controls and named pSiRPG. The downregulation of TFPI was confirmed both at the mRNA and protein level using quantitative RT-PCR and total TFPI ELISA, respectively, as previously described [15]. The self-designed Taqman assays TFPIα (fwd primer: 5'-CAAGAATGTCTGAGGGCATGTAAA-3', rev primer: 5'-CTGCTTCTTTCTTTTTCTTTTGGTTT-3', probe: 5'-AGGGTTTCATCCAAAGAATATCAAAAGGAGGCC-3') and TFPIβ (fwd primer: 5'-CAAGGTTCCCAGCCTTTTTGT-3', rev primer: 5'-CTTGGTAAATATGAGCCGCATTC-3', probe: 5'-TCCAACCATCATTTGTTCCTTCTTTTGT-3') were used for mRNA quantification of the individual isoforms.

### Self-sufficient growth

To study the cells ability to grow when seeded at low densities, transduced Sum102 cells (0.2, 5, and 10·10^3^) were seeded in 12-wells trays and grown for minimum 8 days without reaching confluence. Cells were washed once with phosphate buffered saline (PBS), trypsinized and counted using Nucleocounter (Chemometec, Alleroed, Denmark). In colony formation experiments, 6-wells trays were pre-coated with a 0.6% agar/medium mixture before 2.5·10^4 ^cells/well were seeded individually in 1.7 mL medium containing 0.35% agar. Cells were grown for 4-6 weeks until colonies appeared. Cells were incubated 1 hour with 100 μL of MTT for visualization before trays were scanned using a computer attached scanner (HP Scanjet 8200). Colonies were counted using the image processing and analysis program ImageJ version 1.4.3.67 (Rasband W; National Institute of Health, Bethesda, MD, http://rsbweb.nih.gov/ij/index.html).

### Adhesion and cell spreading

Transduced cells were starved over night in medium without supplements (Sum102) or with 1% FBS (MDA-MB-231) and seeded (5/2.5·10^4^; Sum102/MDA-MB-231) in 96-wells trays coated with fibronectin (10 μg/mL), laminin (10 μg/mL), collagen I or IV (10 μg/mL), vitronectin (0.5 μg/mL), or gelatin (2%). Plates were incubated for 30 minutes and washed gently once with growth medium. Adhered cells were counted indirectly by incubation in medium containing 10 μL WST-1 for 30 minutes followed by analysis at wavelengths 450 and 745 nm in a colorimetric plate reader (Benchmark Microplate Reader, BIO-RAD, Hercules, CA).

For cell spreading analyses, transduced cells were starved over night and seeded (3/1.5·10^5^; Sum102/MDA-MB-231) on glass cover slips coated with fibronectin (10 μg/mL), laminin (10 μg/mL) or collagen I (10 μg/mL), and incubated for 1 hour at 37°C. Cells were washed once in PBS, fixed in 4% formaldehyde in PBS for 15 minutes at room temperature and washed three times in PBS. 0.1% Triton in 100x PBS was added, and after 4 minutes the cells were washed three times in PBS. Subsequently, the cells were dyed with 1% rhodamine-conjugated phalloidin (Invitrogen) for 30 minutes and washed twice in PBS and once in water before the cover slips were mounted on object glasses using flueric mounting medium (Vector Laboratories Inc., Burlingame, CA). Labeled actin was visualized using a fluorescence Nikon eclipse TE300 inverted microscope with a Plan Fluor ELWD 40x/0.60 NA objective (Nikon, Tokyo, Japan) and appropriate filters. Images were captured using a Nikon DS-5M-L1 Camera system. The cell spreading was quantified by measuring the area of the cells using ImageJ.

### Scratch-wound assay

Transduced Sum102 cells were seeded in 6-wells trays and starved over night in medium without supplements. A wound was made in the confluent cell monolayer using sterile pipette tips and the cells were washed gently once with PBS before adding culture medium containing 5% serum. Images were captured after 0, 7.5, and 17 hours using a Nikon eclipse TE300 inverted microscope with a Plan 10x/0.25 NA objective (Nikon) and an attached Nikon DS-5M-L1 Camera system. The width of the wound was measured manually at each time point.

### Cell migration and invasion

Migration of transduced Sum102 and MDA-MB-231 cells was assessed by a modified Boyden`s chamber method using a 24-wells format with transwell Falcon HTS Fluoro Blok inserts (8-μm pore size; Becton Dickinson, Franklin Lakes, NJ, USA). Cells were starved over night in medium without supplements (Sum102) or with 1% FBS (MBA-MB-231), detached with 5 mM EDTA in PBS and seeded (10/5·10^4^; Sum102/MDA-MB-231) in 500 μL of starvation medium in the upper chambers of the transwell. 750 μL culture medium with 10% FBS was added to the lower chambers as a chemoattractant, and the cells were allowed to migrate for 24/16 hours (Sum102/MDA-MB-231) at 37°C. After incubation, the medium was removed and inserts washed once in Hank's Balanced Salt solution (HBSS). Cells were stained with Calcein AM for 90 minutes and migrated cells were detected using a bottom-reading fluorescence plate-reader (Wallac Victor 1420, Perkin Elmer, Waltham, MA) at excitation/emission wavelengths of 485/535 nm.

Invasion of transduced Sum102 and MDA-MB-231 cells was assessed as described for the migration assay, except for the usage of matrigel pre-coated inserts (BD BioCoat Tumor invasion system, Bedford, MA, USA). Cells were incubated for 48/24 hours (Sum102/MDA-MB-231) before labeling. For MMP inhibition experiments, doxycycline hyclate (6 μg/mL, Sigma-Aldrich, St. Louis, MO) was added to the upper chambers directly after cell seeding.

### Protein assay

Transduced cells were seeded to reach 80% confluency after thee days and harvested as previously described [15]. TFPI antigens were measured in the medium and lysate of cells with the commercial Asserachrom^® ^Total and Free TFPI ELISA kits (Diagnostica Stago, Asnière, France), which detect both isoforms of TFPI or only TFPIα, respectively. Urokinase plasminogen activator (uPA) antigen levels and plasminogen activator inhibitor 1 (PAI-1) activity were measured in the medium of cells using the commercial ELISA kits Zymutest uPA antigen (Hyphen BioMed, Neuville-sur-Oise, France) and Trinilize PAI-1 Activity (Trinity Biotech, Jamestown, NY), respectively. Results were corrected against cellular total protein measured in the cell lysate.

MMP-2 and MMP-9 activity in conditioned medium collected from the transduced cells were determined by gelatin zymography. Sum102 (3.25·10^5^) and MDA-MB-231 (4·10^5^) cells were seeded in 6-wells trays and grown for three days before medium was collected and cells lysed in Tris (tris(hydroxymethyl)aminomethane) lysis buffer [15] for Western blot analysis of α-tubulin. For MDA-MB-231 cells, growth medium was replaced with serum-free AIM-V (Invitrogen) medium the day after seeding. The serum-free media were separated on 7.5% polyacrylamide gels containing 0.1% gelatin (type A from porcine skin, Sigma-Aldrich). After electrophoresis, gels were washed twice for 20 minutes in 2.5% Triton X-100 and incubated overnight at 37°C in 50 mm Tris (pH 7.5), 200 mm NaCl, 5 mm CaCl_2 _and 0.02% Brij 35 (Sigma-Aldrich). Positive controls for MMP-2 (recombinant, R&D Systems, Abingdon, UK) and MMP-9 [31] were run with each gel. The gels were stained with 0.1% Coomassie Brilliant Blue R-250 and clear bands indicated gelatinolytic activity.

### PI-PLC treatment

Transduced Sum102 and MDA-MB-231 cells (5·10^6^) were distributed into Eppendorf tubes and washed twice with PBS. Cells were resuspended in 0.55 ml PBS or PBS containing phosphatidylinositol-phospholipase (PI-PLC, 0.7 U/mL, Sigma-Aldrich) and incubated for 2 hours at 37°C. After incubation the cells were pelleted and the supernatant collected. Cell pellets were washed twice in cold PBS and lysed. Cellular total protein was measured in the cell lysate and total and free TFPI antigens were measured in the supernatant as described above.

### Western blot analysis

Transduced Sum102 and MDA-MB-231 cells were starved for 5.5 hours in medium without supplements and FBS, respectively. 1-2·10^6 ^cells were distributed into Eppendorf tubes and stimulated with growth medium containing 10% FBS for 0-60 minutes. Cells were washed twice in PBS and lysed in sample buffer containing 10% DTT, 0.25% Benzonase, 0.2% aprotinin, 0.6% 100 mM PMSF, and 1.0% phosphatase inhibitor cocktail and stored at -20°C until use. The cell lysates were incubated at 97°C for 5 minutes before separated on SDS-polyacrylamide gels (Bio-Rad Laboratories, Hercules, CA). Membranes were incubated with primary antibody over night at 4°C and 1 hour at room temperature with the appropriate horseradish peroxidase (HRP) -conjugated secondary antibody. HRP signals were developed using the ECL Plus Western blotting detection system (GE Healthcare Bio-Sciences, Uppsala, Sweden). The following human antibodies were used; Anti-phospho-Akt (Ser473, #9271), anti-phospho-IκB (Ser32, #2859), anti-phospho-NF-κB (Ser468, #3039), anti-phospho-44/42 MAPK (Thr202/Tyr204, #4377), anti-phospho-SAPK/JNK (Thr183/Tyr185, #9251), and anti-phospho-Src family (Tyr416, #2010) from Cell signaling Technology (Denver, MA), anti-phosphotyrosine HRP conjugate (clone 4G10, #16-105) from Millipore (Billerica, MA), anti-phosphotyrosine PY99 (sc-7020) from Santa Cruz Biotechnology (Santa Cruz, CA), and anti-CD49b (integrin α2, 611016) from BD Biosciences (San Jose, Ca). Anti-c-Src (sc-8056, Santa Cruz Biotechnology) and anti-α-tubulin (#T5168, Sigma-Aldrich) were used as loading controls.

### Statistical analysis

Statistical differences between data from cells with TFPI downregulated and empty vector pSiRPG control cells were calculated using Graphpad Prism 5.0 (Graphpad, San Diego, CA, USA). The unpaired student's t test was used after verification of the normal distribution of the data. The Welch's correction (for unequal variances) was applied when appropriate. For data not normally distributed, the non-parametric Mann-Whitney test was used. The statistical differences in cell migration at 7.5 and 17 hours and in invasion of untreated and doxycycline-treated cells were calculated using two-way ANOVA with Bonferroni corrected post-tests. A probability value (*p*) of < .05 was considered significant.

## Results

### Stable cell lines with TFPI downregulated

Stable cell lines were established through viral transduction and the results of the downregulation are presented in Tables 1 and 2. To measure TFPI antigen levels we used two ELISA assays detecting both isoforms of TFPI (total TFPI ELISA) and only TFPIα (free TFPI ELISA). A 30-79% decrease in total TFPI antigen was measured in the medium of either cell types and corresponding reductions in mRNA levels of both isoforms of TFPI were observed (Table 1). In MDA-MB-231 cells, downregulation of only the β isoform resulted in a 42-46% decrease in TFPIβ mRNA and a 34-43% decrease in total TFPI antigen in the cell lysate. No decrease in TFPIα mRNA or antigen levels was measured (Table 2). An increase in total TFPI antigen was detected in the cell supernatant after treatment with the GPI-cleaving enzyme PI-PLC, compared to untreated cells, and 50% less TFPI was released in the knockdown cells than in empty vector control cells (Tables 1 and 2). No increase in TFPIα antigen was detected after PI-PLC treatment (Tables 1 and 2). The mRNA expression ratio of TFPIβ/TFPIα was 7% in Sum102 wild type cells and 2% in MDA-MB-231 wild type cells (results not shown).

**Table 1:** Stable downregulation of both isoforms of TFPI in Sum102 and MDA-MB-231 breast cancer cells.

		TFPIα/β mRNA **downregulation**, % of control	Total TFPI ag	**Total TFPI ag**, PI-PLC/ Untreated	**Free TFPI ag**, PI-PLC/ Untreated
Sum102 [15]	pSiRPG-shRNA4	60/51	13.0		
	pSiRPG-shRNA6	35/36	28.1	1.3/n.d	n.d/n.d
	pSiRPG-shRNA7	46/42	19.0	1.1/n.d	0.1/n.d
	pSiRPG (control)	0/0	56.7	2.4/n.d	0.1/0.1
					
MDA-MB-231	pSiRPG-shRNA4	62/58	3.6		
	pSiRPG-shRNA6	26/36	12.0	0.7/0.2	0.1/0.2
	pSiRPG-shRNA7	41/46	8.2	0.4/0.2	0.1/0.1
	pSiRPG (control)	0/0	17.2	1.5/0.3	0.2/0.2

Sum102 and MDA-MB-231 breast cancer cells were transduced with vectors containing shRNAs against TFPI or empty vector pSiRPG as a control. Relative TFPIα and β mRNA levels were quantified using qRT-PCR and secreted protein (ag) with total TFPI ELISA kit. GPI attached TFPI was measured in the supernatant after PI-PLC treatment using total and free TFPI ELISA kits (n.d = not detected). Ag levels were corrected against cellular total protein and presented as ng TFPI/mg total protein.

**Table 2:** Stable downregulation of TFPIβ in MDA-MB-231 breast cancer cells.

		TFPIβ/α mRNA downregulation, % of control	Total/Free TFPI ag	Total TFPI ag, PI-PLC/Untreated	**Free TFPI ag**, PI-PLC/Untreated
MDA-MB-231	pSiRPG-shRNA7β	46/2	2.0/1.6	0.7/0.2	0.1/0.1
	pSiRPG-shRNA9β	42/1	2.3/1.6	0.8/0.2	0.1/0.1
	pSiRPG (control)	0/0	3.5/1.7	1.5/0.3	0.2/0.2

MDA-MB-231 breast cancer cells were transduced with vectors containing shRNAs against TFPIβ or empty vector pSiRPG as a control. Relative TFPIα and β mRNA levels were quantified using qRT-PCR and cell-associated protein (ag) with total and free TFPI ELISA kits. GPI attached TFPI was measured in the supernatant after PI-PLC treatment using total and free TFPI ELISA kit. Ag levels were corrected against cellular total protein and presented as ng TFPI/mg total protein.

### Cell growth and colony formation

**Figure 1:**
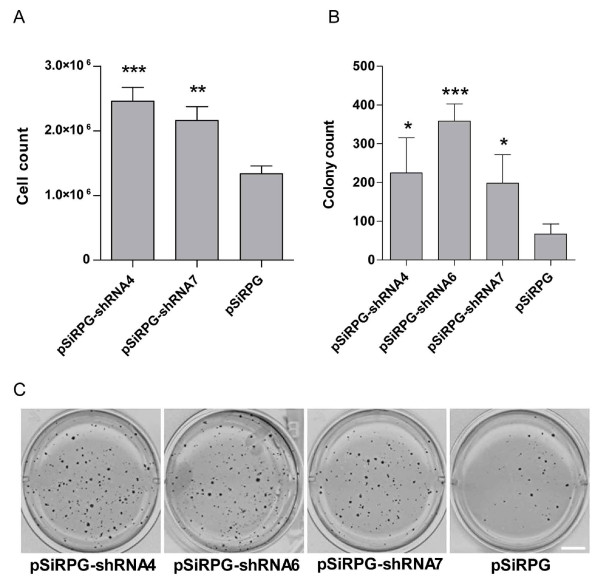
**Self-sustained growth of Sum102 cells after downregulation of TFPI**. (A) Sum102 cells expressing shRNA4 or -7 against TFPI or pSiRPG empty control were seeded at 10000 cells/well in 12-wells trays and grown for a minimum of 8 days. The results are presented as mean cell count (n = 9) + SEM of three independent experiments (**p = .0039 and ***p = .0003 against pSiRPG). (B and C) For colony formation assays, transduced Sum102 cells were seeded individually in soft-agar and grown for 2 to 4 weeks. Colonies were counted using ImageJ and the results are presented as mean number of colonies (n = 3) + SD of one representative experiment (*p < .046 and ***p = .0006 against pSiRPG). Scale bar = 1 cm.

To explore the possible role of TFPI in cancer cell growth, we investigated whether downregulation of TFPI would affect the cells ability to stimulate self-sustained growth. Cells were seeded at very low concentrations (200-10000 cells/well) and their growth rate was evaluated. The Sum102 cells expressing shRNAs against TFPI grew significantly better when seeded at 10000 cells/well than empty vector control cells, counting 60-80% more cells at the day of harvest (Figure 1A). Similar results were observed when 500 cells/well were seeded, while cells seeded at a density of 200 cells/well died (data not shown). We next investigated if downregulation of TFPI would affect the cells ability to form colonies in soft-agar. The results showed that Sum102 cells with TFPI downregulated formed larger and more than twice as many colonies than the empty vector control cells (Figure 1B and 1C). No significant increase in growth rate or colony formation was observed in the MDA-MB-231 cells with TFPI downregulated (data not shown).

### Effect of downregulation of TFPI on cell motility

**Figure 2:**
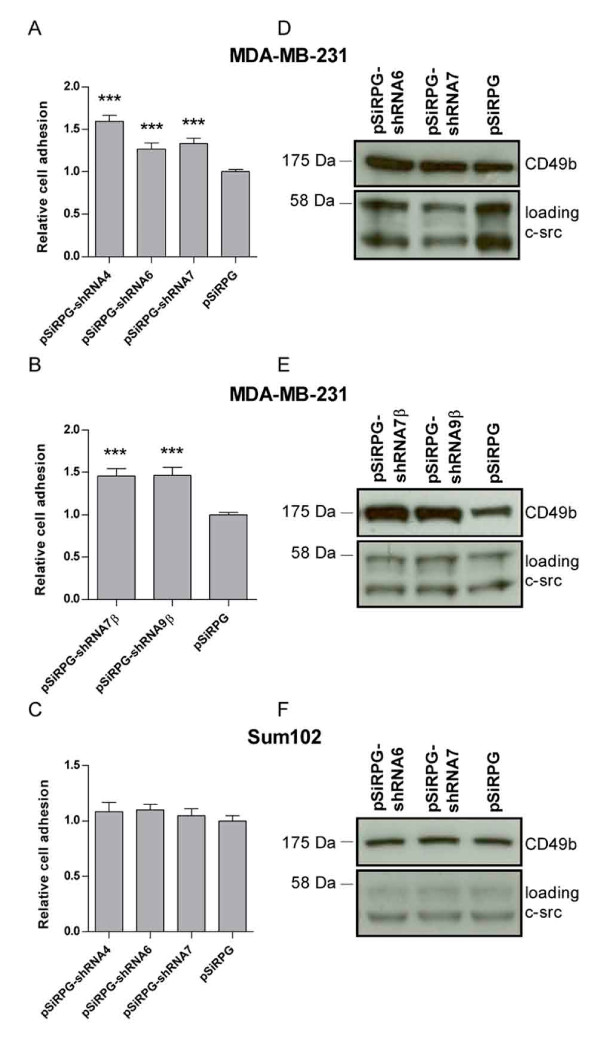
**Adhesion of cells to collagen I and integrin α2 expression**. Transduced cells were seeded in collagen I coated 96-wells trays and incubated for 30 minutes before attached cells were analyzed using WST-1. Results are presented as mean relative adhesion + SEM of three to four independent experiments. (A) MDA-MB-231 cells expressing shRNAs against TFPI and empty vector control (n = 24, ***p ≤ .001 against pSiRPG). (B) MDA-MB-231 cells expressing shRNAs against TFPIβ and empty vector control (n = 18, ***p < .0001 against pSiRPG). (C) Sum102 cells expressing shRNAs against TFPI and empty vector control (n = 18). Integrin α2 protein levels were measured in the total lysate of MDA-MB-231 cells with TFPI (D) and TFPIβ (E) downregulated, and of (F) Sum102 cells with TFPI downregulated using Western blot analysis. c-Src was used as loading control. One representative experiment of two to three is presented.

The increase in self-sustained growth observed in Sum102 cells with TFPI downregulated may also indicate enhanced metastatic growth of these cells. Cell motility was investigated by measuring adhesion, migration, and invasion of the transduced cells. For cell-matrix adhesion experiments, cells were seeded in wells coated with different ECM components that are ligands to various integrin receptors. A significant increase (27-59% and 46%) in adhesion to collagen I was observed in MDA-MB-231 cells with TFPI or TFPIβ downregulated, respectively (Figure 2A and 2B). No significant increase in adhesion of Sum102 cells with TFPI downregulated was detected (Figure 2C), and no differences in the adhesion of either cell types to the ECM components laminin, fibronectin, vitronectin, collagen IV or gelatin were observed in the transduced cells (data not shown). The integrin receptor α2β1 is able to bind to collagen I [32]. To examine the possible involvement of this receptor in the enhanced adhesion observed, the α2 subunit levels were analyzed by Western blot. An increase in integrin α2 protein was seen in the lysate of MDA-MB-231 cells with TFPI (Figure 2D) or TFPIβ (Figure 2E) downregulated, while no difference in integrin α2 levels was observed in the transduced Sum102 cells (Figure 2F).

**Figure 3:**
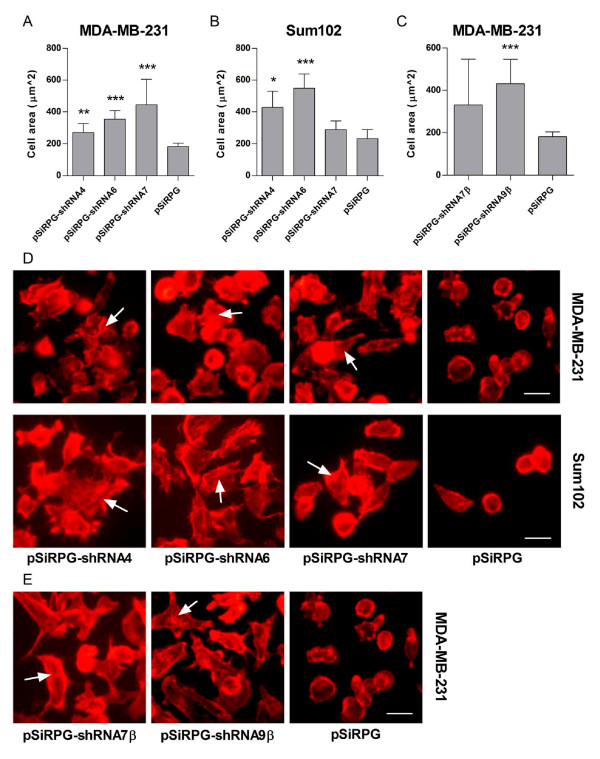
**Cell spreading to collagen I**. Transduced cells were incubated on collagen I coated glass cover slips, fixed and treated with the F-actin binding protein phalloidin. Images were retrieved using a fluorescence Nikon eclipse TE300 inverted microscope with a Plan Fluor ELWD 40x/0.60 NA objective (Nikon Tokyo, Japan) attached to a Nikon DS-5M-L1 Camera system. The cell spreading was quantified by measuring the area of individual cells in a randomly picked field using ImageJ. Results are presented as mean cell area + 95% CI of one representative experiment of two to three. (A) MDA-MB-231 cells (n ≥ 19, **p = .0017 and ***p < .0001 against pSiRPG) and (B) Sum102 cells (n ≥ 5, *p = .0137 and ***p < .0001 against pSiRPG) with TFPI downregulated. (C) MDA-MB-231 with TFPIβ downregulated (n ≥ 19, ***p < .0001 against pSiRPG). (D) Visualization of the actin stress fibers in MDA-MB-231 (upper panels) and Sum102 (lower panels) cells expressing shRNA4 (left), -6 (middle left), and -7 (middle right) against TFPI or pSiRPG control vector (right) and in (E) MDA-MB-231 cells expressing shRNA7β (left) and -9β (middle) against TFPIβ or empty control vector (right). Scale bar = 20 μm.

Next we investigated if downregulation of TFPI affected cell spreading on collagen I coated glass cover slips. The results showed that both MDA-MB-231 and Sum102 cells with lowered TFPI expression were more extended than control cells (Figure 3A and 3B, respectively). An increase in the formation of actin stress fibers was also apparent in MDA-MB-231 (upper panels) and Sum102 (lower panels) cells expressing shRNA4 (left), -6 (middle left) and -7 (middle right) against TFPI compared to empty vector control cells (right) (Figure 3D, white arrows). Similar results were observed when only TFPIβ was downregulated. As seen in Figures 3C and 3E (white arrows), cells were more extended compared to the empty vector control cells and actin stress fibers were clearly visible in cells expressing shRNA7β (left panel) and -9β (middle panel) against TFPIβ compared to control cells (right panel).

**Figure 4:**
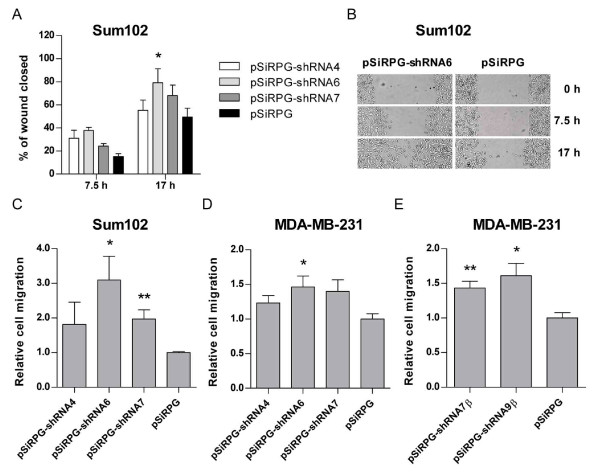
**Migration of transduced cells**. (A) Scratch wound migration of Sum102 cells expressing pSiRPG-shRNA4 (white bars), -6 (light gray bars), -7 (dark gray bars) or control vector pSiRPG (black bars). Images were captured using a Nikon eclipse TE300 inverted microscope with a Plan 10x/0.25 NA objective (Nikon) and an attached Nikon DS-5M-L1 Camera system. Results are presented as the average width of the wound (n = 4) + SEM of one representative experiment of two (*p <.05 against pSiRPG). (B) Scratch wound widths after 0, 7.5 and 17 hours in cells expressing shRNA6 against TFPI (left) or pSiRPG control vector (right). Migration of transduced cells was also measured using the modified Boyden method. Cells were incubated for 24/16 hours (Sum102/MDA-MB-231) at 37°C before stained with Calcein AM for 90 minutes. Results are presented as mean relative migration + SEM of three to four independent experiments. (C) Sum102 cells (n ≥ 8, *p = .0143 and **p = .0051 against pSiRPG) and (D) MDA-MB-231 cells (n = 9, *p = .0177 against pSiRPG) with TFPI downregulated. (E) MDA-MB-231 cells with TFPIβ downregulated (n = 9, *p = .0103 and **p < .0027 against pSiRPG).

To further investigate the effect of downregulation of TFPI on cell motility, we analyzed the cells ability to migrate. In the scratch wound assay, Sum102 cells expressing shRNA4 (white bars), -6 (light gray bars) and -7 (dark gray bars) against TFPI migrated faster into the wound compared to empty vector control cells (black bars) (Figure 4A and 4B). 24-38% of the cell wounds were closed after 7.5 hours of serum stimulation when TFPI was downregulated, compared to 15% in the control cells. After 17 hours, 55-79% of the wounds were closed in the knockdown cells, but only 49% in the controls. The effect of downregulation of TFPI on chemotaxis was also investigated using modified Boyden chambers where cells migrate through pores towards an attractant. Sum102 cells expressing shRNA6 and -7 against TFPI showed a significant 210% and 100% increase in migration, respectively, compared to control cells (Figure 4C). Moreover, a significant 50% increase in migration of MDA-MB-231 cells expressing shRNA6 against TFPI was also observed (Figure 4D). Downregulation of TFPIβ resulted in a significant 40% and 60% increase in MDA-MB-231 cell migration (Figure 4E).

**Figure 5:**
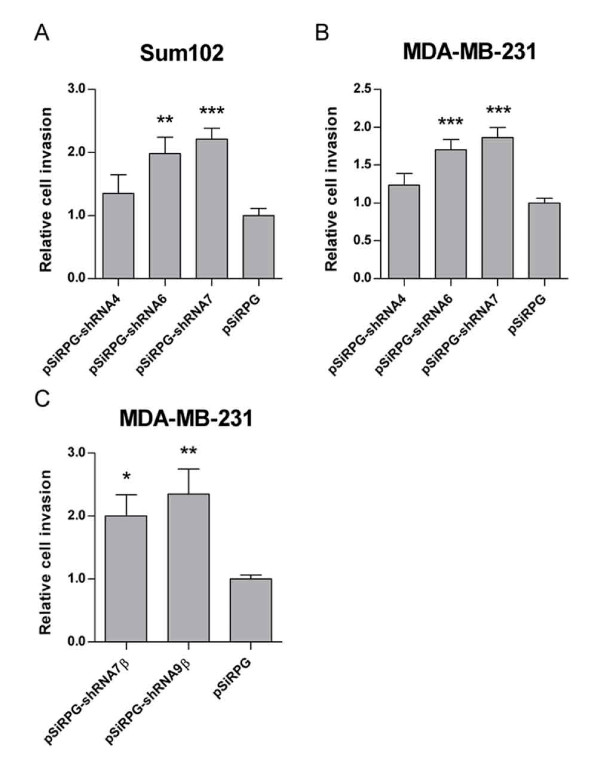
**Invasion of transduced cells measured using modified Boyden chambers**. Cells were seeded in matrigel pre-coated chambers and incubated for 48/24 hours (Sum102/MDA-MB-231) at 37°C before stained with Calcein AM. Results are presented as mean relative invasion + SEM of three independent experiments. (A) Sum102 cells (n ≥ 8, **p = .0063 and ***p ≤ .0001 against pSiRPG) and (B) MDA-MB-231 cells (n ≥ 8, ***p < .0001 against pSiRPG) with TFPI downregulated. (C) MDA-MB-231 cells with TFPIβ downregulated (n ≥ 8, *p = .0227 and **p = .0098 against pSiRPG).

To investigate whether downregulation of TFPI would affect the cells invasive abilities, we used modified Boyden chambers coated with matrigel to mimic the ECM. Compared to the empty vector control cells, a significant 100% and 120% increase in invasion were observed in Sum102 cells expressing shRNA6 and -7 against TFPI, respectively (Figure 5A). In MDA-MB-231 cells expressing shRNA6 and -7 against TFPI, a significant 70% and 90% increase in invasion was detected, respectively, compared to control cells (Figure 5B). A significant 100% and 140% increase in cell invasion was seen when only TFPIβ was downregulated (Figure 5C).

### The plasminogen activator system, MMP activity, and intracellular signaling

**Figure 6:**
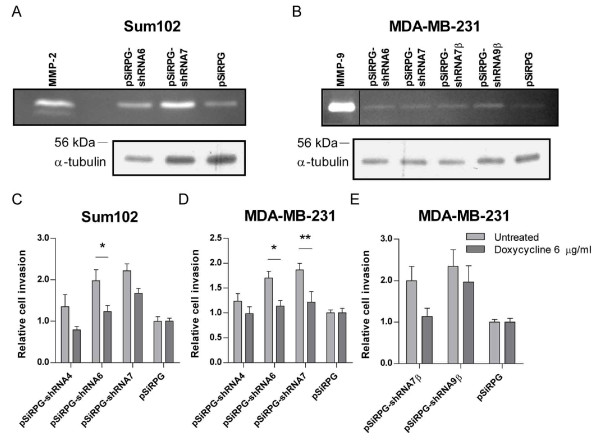
**MMP activity and the effect of the general MMP inhibitor doxycycline hyclate**. MMP-2 and MMP-9 activity in serum-free media of transduced Sum102 (A) and MDA-MB-231 (B) cells, respectively, as determined by gelatin zymography. Transduced cells were seeded in 6-wells trays and grown for three days. Media were replaced with conditioned medium (Sum102) or serum-free AIM-V medium (MDA-MB-231) after 24 hours. α-tubulin was used as loading control. One representative experiment of three is shown. (A lane was removed to make the image smaller in B) The relative invasion of transduced cells untreated (gray bars) or treated with doxycycline hyclate (dark gray bars) was measured using the modified Boyden chamber method. Doxycycline (6 μg/mL) was added to the upper chambers immediately after cell seeding. Results are presented as mean relative invasion + SEM of two to three independent experiments. (C) Sum102 cells (n ≥ 8, *p < .05) and (D) MDA-MB-231 cells (n ≥ 6, *p < .05 and **p < .01) with TFPI downregulated. (E) MDA-MB-231 cells with TFPIβ downregulated (n ≥ 6).

Plasmin and the plasminogen activator system may directly or indirectly affect the migration and invasion of cells [33,34]. To investigate if this system could be responsible for the increased motility observed in cells with TFPI downregulated, the plasmin activator, uPA, and its inhibitor, PAI-1, were analyzed. The results showed no increase in uPA antigen levels or in PAI-1 activity in the medium of the transduced Sum102 and MDA-MB-231 cells (data not shown). MMPs are able to cleave ECM and are therefore important for the invasion of cancer cells [29]. To investigate the possible involvement of these proteases, gelatin zymography analyses were performed. The results showed an increase in MMP-2 activity in Sum102 cells with TFPI downregulated (Figure 6A). In MDA-MB-231 cells, elevated MMP-9 activity was observed when both isoforms of TFPI or just TFPIβ was downregulated (Figure 6B). Insignificant MMP-9 and MMP-2 activity was detected in the Sum102 and MDA-MB-231 cells, respectively. To confirm that MMPs were involved in the increased invasion observed, the general MMP inhibitor doxycycline hyclate was added to the upper chamber in the invasion assays at a non-cytotoxic concentration. The results showed that the relative invasion was reduced by 24-41% following doxycycline treatment of Sum102 cells expressing shRNAs against TFPI. This reduction was significant in shRNA6 expressing cells (Figure 6C, gray bars; untreated, dark gray bars; doxycycline treated). In MDA-MB-231 cells, doxycycline treatment significantly reduced the relative invasion by 34-35% when shRNA6 and -7 were expressed (Figure 6D). In cells with TFPIβ downregulated, a 16-43% reduction in relative invasion was observed (Figure 6E).

**Figure 7:**
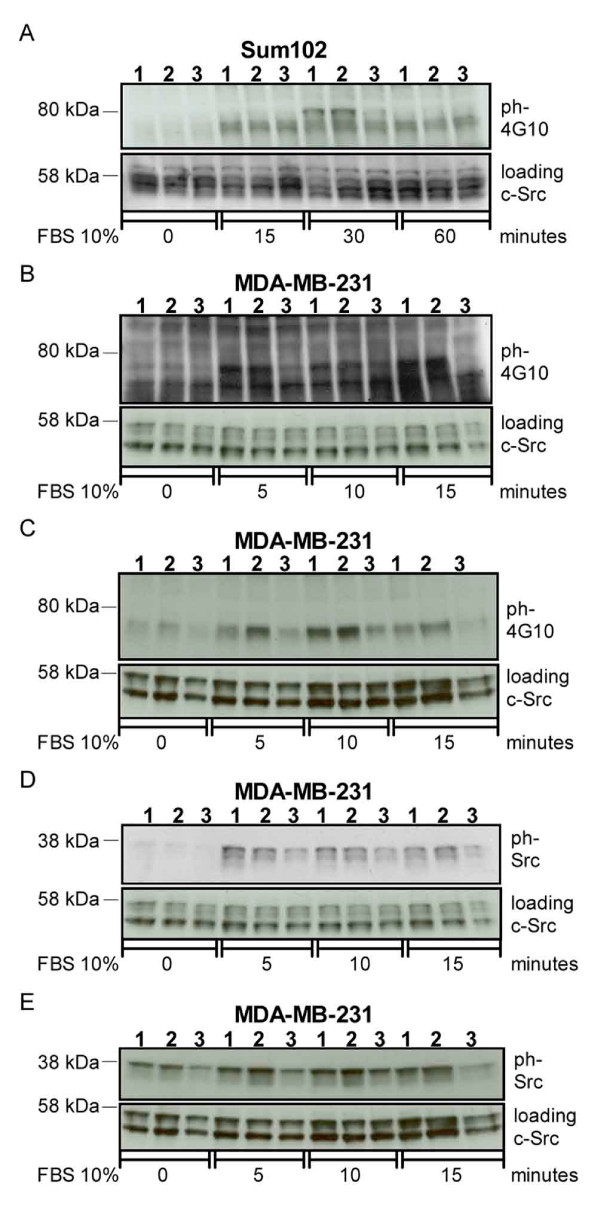
**Phosphorylation status of signaling molecules in the total lysate of transduced cells after FBS stimulation**. Cells were starved for 5.5 hours before 1-2 × 10^6 ^cells were stimulated with 10% FBS for indicated time points. General tyrosine phosphorylation in Sum102 (A) and MDA-MB-231 (B) cells expressing shRNA6 (lane 1), -7 (lane 2) or empty vector control (lane 3) and (C) in MDA-MB-231 cells expressing shRNA7β (lane 1), -9β (lane 2) or empty vector control (lane 3), detected using anti-4G10 antibody. Src family phosphorylation in MDA-MB-231 cells expressing (D) shRNA6 (lane 1), -7 (lane 2) or empty vector control (lane 3) and (E) shRNA7β (lane 1), -9β (lane 2) or empty vector control (lane 3), detected using anti-phospho-Src antibody. c-Src was used as loading control. One representative experiment of three is shown.

The molecular mechanisms responsible for the increase in cell motility observed after downregulation of TFPI were unclear. To investigate this we analyzed the phosphorylation status of several signal transduction molecules known to be involved in these processes. The results showed increased tyrosine phosphorylation of a protein just under 80 kDa in both Sum102 (Figure 7A) and MDA-MB-231 (Figure 7B) cells expressing shRNA6 (lane 1) and -7 (lane 2) against TFPI compared to control cells (lane 3). Similar results were obtained in MDA-MB-231 cells expressing shRNA7β (lane 1) and -9β (lane 2) against TFPIβ compared to control cells (lane 3) (Figure 7C). An increase in tyrosine 416 phosphorylation of a Src family member was also seen in MDA-MB-231 cells with TFPI (Figure 7D, shRNA6; lane 1, shRNA7; lane 2) and TFPIβ (Figure 7E, shRNA7β; lane 1, shRNA9β; lane 2) downregulated compared to empty vector control cells (lanes 3). No increase in the phosphorylation status of Akt, IκB, NFκB, ERK1/2, and SAPK/JNK was observed in the transduced cells at the time points tested (data not shown).

## Discussion

TFPI is mainly recognized for its activity in the coagulation cascade. Previous findings have, however, indicated an additional role of TFPI in cancer biology. We recently showed that shRNA mediated downregulation of TFPI inhibited apoptosis, while ectopic overexpression of TFPI induced apoptosis in breast cancer cells, supporting this hypothesis [15]. In the present study, we demonstrate that stable downregulation of both isoforms of TFPI and TFPIβ alone enhanced the metastatic growth of breast cancer cells by increasing cell spreading, migration and invasion. The phosphorylation status of signaling molecules was affected and elevated MMP-2 and MMP-9 activity was observed that could explain the increased cell invasion. These results further supports an anti-tumor effect of TFPI and indicate a new role of TFPIβ in cancer motility.

The effects of downregulation of TFPI were investigated in the two basal-like invasive breast carcinoma cell types Sum102 and MDA-MB-231. These cells are derived from a primary and a metastatic tumor, respectively, and both express endogenous TFPI; the Sum102 cells approximately four times more than the MDA-MB-231 cells (Table 1). Stable downregulation of TFPI was conducted using RNA interference, and shRNAs targeting both isoforms of TFPI or only TFPIβ, were used. In the MDA-MB-231 cells with TFPIβ downregulated, no decrease in TFPIα antigen was measured verifying that the knockdown was isoform specific. PI-PLC treatment of the transduced cells resulted in the release of TFPI from the cell surface. This was only detectable when total TFPI antigen was measured and confirmed that TFPIβ was attached to the cell surface through a GPI anchor. Also, less TFPI was released in the knockdown cells than in the control cells, which showed that TFPIβ was downregulated at the protein level. The functional effects observed in this study following downregulation of TFPI did not appear to be dose-dependent.

Sum102 cells with TFPI downregulated grew better when seeded at low densities and formed more colonies than control cells. This indicated an increased ability to stimulate self-sufficient growth, both dependent and independent of cell anchorage. Self-sufficient growth signaling often involves changes in expression or activity of growth factors, receptors and signaling molecules in the cells [25]. No increase in cell growth was observed when empty vector control cells were treated with medium taken from knockdown cells seeded at low density (results not shown). This could indicate that the increase in growth signaling was not due to elevated secretion of growth factors but rather to enhanced expression or activity of cell surface receptors and/or signaling molecules. The induced intracellular signaling observed in cells with TFPI downregulated may support this. No effect of downregulation of TFPI was observed on the self-sustained growth of MDA-MB-231 cells in this study. The reason for this is unclear although the two cell types Sum102 and MDA-MB-231 grow at different rates.

The increase in adhesion and stress fiber formation observed in MDA-MB-231 cells to an ECM component indicated that downregulation of TFPI enhanced the integrin-mediated adhesion and -cell spreading of these cells. Moreover, as the increase in adhesion and cell spreading was specific to collagen I, and not to the other ECM components tested, it implied that downregulation of TFPI resulted in enhanced expression and/or activity of collagen I-binding integrins, such as α_2_β_1 _[32]. No difference in adhesion to collagen I was measured in the transduced Sum102 cells, although the cells were more extended and an increase in actin stress fiber formation were observed when TFPI was downregulated. This indicated an affect on integrin-mediated cell spreading only. Enhanced protein expression of the α2 integrin subunit was only found in MDA-MB-231 cells after downregulation of TFPI, which may explain the increased adhesion observed in these cells. Provencal and coworkers [35] previously reported that recombinant TFPI (rTFPI) had no effect on adhesion of U-87 glioblastoma cells to gelatin, which is in line with our results (data not shown). Moreover, in this study rTFPI was reported to have no effect on the migration of brain cancer cells, when the membrane was coated with gelatin.

The molecular mechanisms and processes responsible for the increase in motility observed in cells with TFPI downregulated are not known. The elevated MMP-2 and MMP-9 activity measured in these cells indicated that such proteases are involved in the increased cell invasion detected. Enhanced MMP activity does not, however, explain the increase in adhesion, cell spreading, and migration observed following downregulation of TFPI, and no evidence for the involvement of the plasminogen activator system was found. Thus, other processes not investigated in this study must have been affected. The increase in phosphorylation of an 80 kDa protein and a Src kinase observed in cells with TFPI downregulated may indicate that these signal transduction pathways were involved. TF has been shown to promote tumor cell migration, invasion, growth, and metastasis [36-40], and Hembrough and colleagues [16] reported that TFPI suppressed metastatic tumor growth *in vivo *through inhibition of TF/FVIIa activity. Both cell types used in the present study express TF [15,36], and a possible enhanced TF activity could therefore be responsible for the increased cell motility observed after downregulation of TFPI. In a chromogenic substrate FXa activity assay indirectly measuring cell surface TF activity, we detected an 80-150% increase in TF activity in Sum102, but not in MDA-MB-231, cells with TFPI downregulated (data not shown). This may indicate that elevated TF activity could have been involved in the increased motility of Sum102 knockdown cells. We were, however, unable to detect activation of the MAP kinase cascade in these cell, which is a common downstream effect of TF/FVIIa induced intracellular signaling [41]. It has previously been shown that treatment of brain cancer cells with rTFPI did not affect the phosphorylation status of the MAP kinases ERK1/2 [35], which is also consistent with our findings.

TFPIα and TFPIβ are the main splice variants transcribed from the TFPI gene but the function of the TFPIβ isoform remains unclear [14]. Surprisingly, downregulation of only TFPIβ in MDA-MB-231 cells resulted in increased cell motility. We recently reported that overexpression of TFPIβ induced apoptosis in SK-BR-3 breast cancer cells [15], but no effects of TFPIβ on cancer cell motility have previously been published. Our findings therefore support a novel role of TFPIβ in cancer cell metastasis. Compared to TFPIα, TFPIβ lacks the third Kunitz domain and has a different C-terminal end containing a GPI anchor. The importance of the C-terminal part of TFPIα in non-hemostatic activities has previously been reported both *in vivo *[24] and *in vitro *[18-24], and TFPIα has been shown to be able to inhibit cell proliferation by binding to the very low density lipoprotein receptor (VLDLR) through its Kunitz-3 and C-terminal domains [19]. The effects on cell motility observed in the present study after downregulation of TFPIβ indicated, however, that additional mechanisms were involved since TFPIβ lacks the Kunitz-3 domain and has a different C-terminal end than TFPIα. The two isoforms of TFPI share the Kunitz-1 and Kunitz-2 domains and it therefore seems plausible that TFPI induced the anti-tumor effects through association with an unknown receptor, involving the first two Kunitz domains, resulting in the tyrosine phosphorylation of an ~75 kDa protein. However, the similar functional effects observed following downregulation of TFPIβ alone and of both TFPIα and TFPIβ may also indicate that TFPIβ was responsible for most of the effects seen in our study. This was further supported by the low TFPIβ/TFPIα ratio observed in these cells. It is evident that TFPIβ is directly attached to the cell membrane through a GPI anchor (Table 1 and 2, [13]). GPI anchored proteins are known to be concentrated in areas of the plasma membrane recognized as lipid rafts [42,43], and the GPI anchor has been reported to be implicated in signal transduction [44]. The Src protein family may also associate with these microdomains and these kinases have been shown to be involved in cell signaling stimulated by GPI anchored molecules [45]. Furthermore, endothelial GPI-bound TFPI has previously been localized to caveolae [46], which are invaginated lipid rafts [47]. This may indicate that the effect of TFPIβ on cancer cell motility observed in this study could have been mediated through its GPI anchor.

## Conclusions

In summary, downregulation of TFPI induced intracellular tyrosine signaling and increased the self-sustained growth and metastatic abilities of breast cancer cell lines *in vitro*. Increased signal transduction and cell motility was also observed after downregulation of just TFPIβ suggesting a new role for this isoform in cancer metastasis. These results may contribute to new therapeutic approaches in cancer.

## Competing interests

The authors declare that they have no competing interests.

## Authors' contributions

MS, NI, MT, LZ, and BS performed experiments; BS designed experiments, analyzed results and wrote the paper; H-CÅ provided and interpreted western blot analyses and edited the paper; GS, PMS and NI planned the project, interpreted results and edited the paper; NI designed the research. All authors have read and approved the final manuscript.

## Pre-publication history

The pre-publication history for this paper can be accessed here:

http://www.biomedcentral.com/1471-2407/11/357/prepub
